# Spathology: The Birth of Spatial Pathology as a Discipline

**DOI:** 10.3390/life16101680

**Published:** 2026-10-08

**Authors:** Mengping Long, Mats Nilsson, Taobo Hu

**Affiliations:** Science for Life Laboratory, Department of Biochemistry and Biophysics, Stockholm University, 171 65 Solna, Sweden

**Keywords:** spatial pathology, spathology, spatial transcriptomics, spatial proteomics, computational pathology, digital pathology, multiplexed imaging, tumor microenvironment, biomarker discovery, artificial intelligence

## Abstract

Pathology has always been a spatial science: for more than 150 years, diagnosis has rested on reading the architecture of cells and tissues. Three technological streams are now converging on this founding principle. Spatially resolved omics can measure thousands of transcripts and over a hundred proteins in intact, formalin-fixed paraffin-embedded (FFPE) tissue; multiplexed imaging quantifies tissue microenvironments cell by cell; and computational pathology has progressed from slide digitization to foundation models that can predict molecular features from morphology. The clinical evidence for spatial readouts is real but heterogeneous: a few assays have reached guideline mention or prospective use, while most findings remain retrospective, platform-dependent, and unevenly validated. Yet the field remains fragmented across “spatial biology”, “digital pathology”, and “computational pathology”, without a unified framework for standards, training, and diagnostics. Here we propose, for discussion rather than as a settled matter, that this convergence be treated as a coherent branch of pathology, “spatial pathology”, which we define as the use of spatially resolved measurements of tissue to derive diagnostic, prognostic, and predictive information. We set out which problems existing structures do not yet address, acknowledge the counterarguments, and outline a consensus-building agenda. We also suggest “spathology” as an optional shorthand, and we disclose openly that all authors are co-founders of a company, SPATHO AB, whose name derives from the same contraction.

## 1. Pathology Has Always Been Spatial

Modern pathology was founded on a spatial claim. Virchow’s cellular pathology located disease in cells and in the tissue architecture they compose, to be read under the microscope [1]. Much of what is durable in diagnostic practice since then has been, at its core, codified spatial pattern recognition: Gleason grading scores the architectural growth patterns of prostate cancer [2]; TNM staging formalizes the anatomic extent of disease [3]; HER2 immunohistochemistry is scored by the completeness, intensity, and distribution of membrane staining across tumor cells [4]. Even immunohistochemistry itself—descended from the first fluorophore-conjugated antibody of 1941 [5]—is a spatial molecular assay, albeit one that has remained essentially single-plex in the clinic for eight decades.

**Figure 1:**
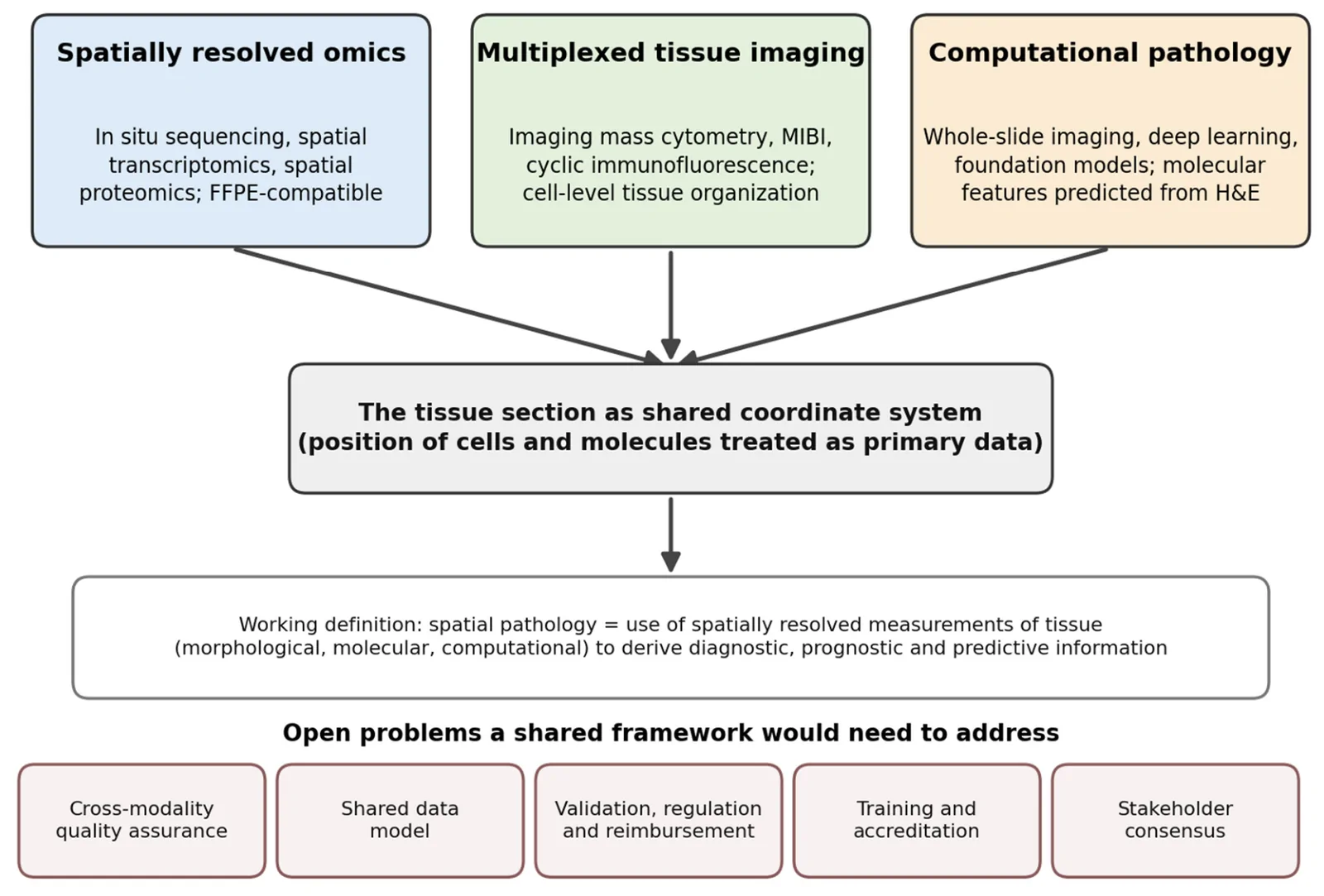
Three technological streams—spatially resolved omics, multiplexed tissue imaging, and computational pathology—converge on the tissue section as the shared coordinate system. The working definition of spatial pathology proposed here and the open problems it would need to address are shown below the confluence.

Pathology, in other words, has practiced spatial biology since its founding. What has changed is not the principle but the instrumentation: the number of molecular species that can be measured at each position in a tissue section has grown from one to tens of thousands, and the computational capacity to interpret such measurements has grown with it. Whether this change warrants a shared framework, and possibly a name, for a distinct branch of pathology is a question for the community, not for three authors. In this short opinion article, we set out the case for and against, propose a working definition, and suggest what a consensus process would need to address. Figure 1 summarizes the argument.

## 2. Three Streams, One Confluence

### 2.1. Spatially Resolved Omics

The molecular stream begins with single-molecule fluorescence in situ hybridization, which first rendered individual transcripts visible in fixed cells [6]. In situ sequencing then showed that RNA could be decoded directly inside morphologically preserved tissue, amplified by padlock probes and rolling-circle amplification and sequenced in place; it was demonstrated from the outset in human breast cancer sections, with point-mutation sequencing shown in cultured cells [7]. Combinatorial barcoding scaled imaging-based profiling to as many as a thousand genes per cell [8], while spatial barcoding of capture arrays made unbiased, whole-transcriptome measurement possible in tissue sections and gave the field its name, spatial transcriptomics [9]. Successive generations of array chemistry pushed resolution to the subcellular scale and fields of view to whole organs [10].

Two developments moved these technologies from atlas-building toward the pathology department. The first is commercialization into robust platforms designed around routine specimens—region-of-interest digital spatial profiling [11], cyclic-hybridization imagers measuring ~1000 RNAs or >100 proteins at subcellular resolution [12], in situ sequencing platforms benchmarked directly on clinical tumor material [13]. The second, and arguably decisive, development is compatibility with formalin-fixed, paraffin-embedded (FFPE) tissue [14]: FFPE is the universal substrate of diagnostic practice and of the world’s tissue archives, with decades of outcome annotation attached. By 2024, many major commercial platforms were FFPE-compatible, and whole-transcriptome profiling at single-cell scale is now feasible on routine FFPE sections [15]. The field’s coming of age has been formally recognized twice in five years: spatially resolved transcriptomics was *Nature Methods*’ Method of the Year 2020 [16], and spatial proteomics its Method of the Year 2024 [17]. The technology stack continues to broaden into spatial epigenomics, metabolomics, and three-dimensional approaches [18,19], as well as unbiased mass-spectrometry-based spatial proteomics performed directly on archived diagnostic material [20].

### 2.2. Multiplexed Tissue Imaging

The protein stream extended immunohistochemistry from one marker to dozens. Imaging mass cytometry [21] and multiplexed ion beam imaging [22]—published simultaneously in 2014 and both demonstrated on breast cancer tissue—used metal-tagged antibodies to escape the spectral limits of fluorescence, while DNA-barcoded cyclic immunofluorescence achieved comparable plex on conventional microscopes [23]. These methods did more than add markers: they made the *organization* of tissue quantifiable. Cellular-neighborhood analysis showed that recurrent multicellular structures at the colorectal cancer invasive front stratify patient outcome [24], and single-cell pathology atlases of breast cancer demonstrated that spatially defined tissue phenotypes carry prognostic information beyond conventional subtyping [25]. Deep-learning segmentation trained on more than a million annotated cells now converts such images into single-cell data at human-level accuracy [26]—an early example of the mutual dependence of spatial assays and artificial intelligence.

### 2.3. Computational Pathology

The computational stream is the youngest but has moved the fastest. Whole-slide imaging was authorized for primary diagnosis in the United States in 2017 on the strength of a 1992-case noninferiority trial [27], creating the data substrate for machine learning. Within a few years, deep learning matched or exceeded pathologists on defined diagnostic tasks [28], predicted driver mutations [29] and microsatellite instability [30] directly from hematoxylin-and-eosin (H&E) morphology, reached pathologist-level Gleason grading in blinded international validation [31], and produced an AI tool for assisting prostate cancer detection on biopsy images authorized by the U.S. Food and Drug Administration (FDA) [32]. The current foundation-model era—self-supervised encoders pretrained on hundreds of thousands to over a million whole-slide images [33,34,35]—provides general-purpose representations of tissue morphology that transfer across organs, tasks, and institutions. What these morphology-only models still largely lack is molecular grounding, and what still separates benchmark performance from clinical deployment is prospective validation, generalization, and workflow integration [36].

Recent work applies machine learning directly to spatial omics data. Foundation models pretrained on tens of millions of dissociated and spatially resolved cells learn representations of spatial context and can transfer spatial information to dissociated data, although models trained on dissociated data alone fail to recover the complexity of spatial microenvironments [37]. Open-source frameworks such as Squidpy have made spatial statistics and image-feature analysis routine [38], and new methods predict spatial gene expression at near-single-cell resolution from histology [39,40,41]. Open problems remain. Benchmarks report limited accuracy and spatial resolution for many histology-to-expression methods [40], and predictive performance depends strongly on the sparsity and noise of the underlying molecular data, with limited gains from imputation, as shown in a recent preprint [42].

## 3. What Spatial Readouts Have Shown, and How Mature the Evidence Is

The claim that spatial context carries diagnostic information beyond bulk or single-marker assays rests on examples of very different maturity. Table 1 lists the main ones and, for each, what has and has not been established. We do not claim that spatial readouts are generally superior to non-spatial biomarkers, or that spatial assays are clinically ready; we claim that a small number of spatial biomarkers have been taken further than the rest, and that they show what is possible.

The Immunoscore is the founding example: the type, density, and—critically—*location* of T cells in colorectal cancer predicted outcome better than the histopathological staging systems in use at the time [43]. An international 2681-patient validation study subsequently showed that this digitally quantified, location-aware assay contributed more to recurrence-risk prediction than any conventional parameter, including TNM stage itself, with inter-observer and inter-center reproducibility of r = 0.97 [44]. The European Society for Medical Oncology guideline for localized colon cancer notes that the Immunoscore may be used alongside TNM to refine prognosis, while stating that its role in predicting chemotherapy benefit is uncertain [45]. Tertiary lymphoid structures, organized immune aggregates identifiable on tissue sections, were associated in 2020 with checkpoint-inhibitor benefit in melanoma, renal cell carcinoma, and sarcoma [46,47,48]. One phase 2 sarcoma cohort was then selected on the presence of such structures: the six-month non-progression rate was 40%, compared with 4.9% in earlier unselected cohorts, a cross-cohort comparison from a single small trial [49]. A meta-analysis of 8135 patients reported a higher pooled discrimination for multiplexed, spatially resolved immunohistochemistry/immunofluorescence (area under the curve, AUC, 0.79) than for PD-L1 immunohistochemistry (0.65), gene-expression profiling (0.65), and tumor mutational burden (0.69) for response to PD-1/PD-L1 blockade [50]; the underlying studies were heterogeneous and largely retrospective, and a whole-slide multiplexed platform later reported external validation of spatial predictors in melanoma [51].

Highly multiplexed and spatially resolved studies have gone on to show that tumor–immune architecture, not merely cellular composition, is associated with survival in triple-negative breast cancer [52]; that recurrent microenvironmental structures link genotype to outcome across hundreds of breast tumors [53]; and that recurrent stromal–immune niches, co-localized in spatial data, underpin clinically distinct ecotypes [54]. Spatial profiling has also begun to unsettle diagnostic categories themselves: in ductal carcinoma in situ, the state of the surrounding stroma and myoepithelium—more than tumor-cell features—tracks progression risk, sometimes in unexpected directions [55], and organ-wide spatial transcriptomics of the prostate has revealed clonal copy-number expansions in histologically benign epithelium, challenging the morphological definition of “normal” [56]. Nor is the case confined to oncology: spatial multi-omics has mapped the reorganization of human myocardial infarction [57], and organ-level roadmaps now explicitly co-locate spatial omics and digital pathology as the future of tissue diagnostics [58]. Most of these studies are discovery-stage and retrospective; they motivate assay development rather than replace it.

## 4. The Convergence Loop: Morphology–Molecule Inference

**Figure 2:**
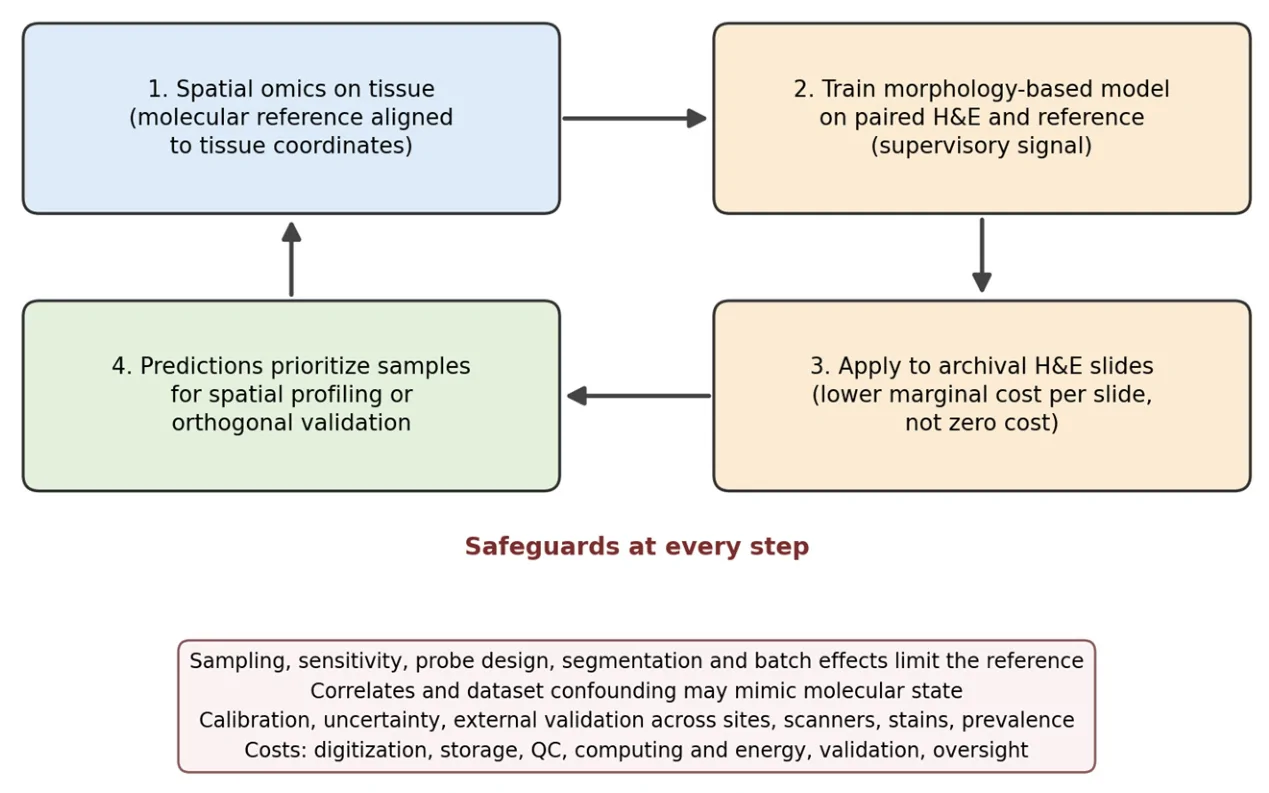
The morphology–molecule loop and its safeguards. Spatial omics provides a molecular reference (a supervisory signal, not an unqualified ground truth); morphology-based models trained on it are applied to archival slides; predictions are used to prioritize samples for orthogonal validation, which feeds back on the reference. Each step carries uncertainty that must be measured and reported.

One specific mechanism links the three streams. Spatially resolved omics can provide a molecular reference, aligned to tissue coordinates, against which morphology-based models are trained; the trained models can then be applied to archival H&E slides, with lower marginal cost per slide than new spatial profiling; and the resulting predictions can help prioritize which samples justify spatial profiling or orthogonal validation, which in turn refines the reference (Figure 2).

This loop is already running. Deep learning trained on spatial transcriptomics spots matched to histology predicted the local expression of more than 100 genes from breast cancer H&E [60]; transcriptome-scale prediction from whole-slide images followed, with “virtual spatialization” of the inferred expression [61]; integration of spatial transcriptomics with high-resolution histology now yields near-single-cell expression inference, including in sections for which only H&E exists [39]; and a visual–omics foundation model aligning histology with spatial transcriptomics has generalized the approach [62].

The loop should not be over-read. Spatial omics measurements are themselves affected by sampling, sensitivity and specificity, probe design, tissue processing, cell segmentation, spatial resolution, batch effects, and platform-dependent error [59]; they are a molecular reference, not ground truth. Predictions of molecular state from H&E may reflect morphological correlates or dataset-specific confounding rather than recovery of the molecular state itself, and performance can fall across institutions, scanners, staining protocols, and disease prevalence [36]. Any use of such predictions beyond research would require calibration, uncertainty estimates, external validation, and explicit attention to causal interpretation. Nor is the marginal cost of applying a trained model to archival slides zero: digitization, storage, quality control, computation and its energy footprint, model validation, integration, and professional oversight all carry costs. The claim is therefore modest: spatial omics and computational pathology are becoming interdependent, which is a reason for the two communities to share standards and vocabulary.

## 5. Is a Shared Framework Needed, and Should It Have a Name?

### 5.1. Which Problems Existing Structures Do Not Yet Address

Technological convergence alone does not establish the need for a new discipline. The relevant question is which scientific, clinical, regulatory, or organizational problems are not adequately addressed within existing pathology structures. We see four candidates.

*Quality assurance across modalities.* External quality assessment exists for immunohistochemistry, for molecular assays, and increasingly for digital pathology, but none spans assays that combine imaging and molecular measurements at spatial coordinates, and platform-to-platform comparisons show substantial discordance [59].

*A shared data model.* Existing pathology information systems handle slides and reports, and genomics handles sequence files; multimodal, coordinate-resolved data sit between them, and the emerging community formats [63] have not yet been integrated with clinical systems.

*Regulatory and reimbursement categories.* Multiparametric spatial assays fit neither the category of a single immunohistochemical marker nor that of a conventional molecular test, and there is no agreed way to specify their analytical and clinical validation.

*Training and accreditation.* Few programs train diagnostic pathologists in coordinate-resolved molecular data, or computational scientists in tissue morphology.

There are reasonable counterarguments. Each of these problems could be addressed inside existing bodies—for example by working groups within digital pathology, molecular pathology, or immuno-oncology societies—and a new label carries the risk of fragmentation and of overstating novelty. Pathology has absorbed some earlier re-instrumentations within existing structures, while others, such as molecular and digital pathology, developed their own societies and training; which path is appropriate here is not obvious. We therefore present a distinct branch of pathology as one option to be weighed, not as a foregone conclusion, and we note that a discipline cannot be established by proposing a name: it requires consensus among practicing diagnostic pathologists, laboratory-medicine and quality-assurance specialists, clinicians, computational scientists, and patient representatives, most of whom are not represented among the authors.

### 5.2. The Name

Terminology may seem a small matter, but the history of our field suggests otherwise. “Telepathology” was coined in a two-page 1986 editorial [64] and matured into practice. “Computational pathology” followed a deliberate arc—technical definition [65], a convened stakeholder meeting that explicitly defined “the scope and needs within the new discipline” [66], and a society white paper fixing terminology and regulatory framing [67]—and is today a recognized field with its own training pathways, meetings, and industry. Other coinages, such as “pathomics”, were introduced without an agreed definition, have been used inconsistently, and have not consolidated into a discipline; some names simply do not take.

“Spatial pathology” is the descriptive term we propose. The exact phrase has appeared only sporadically in the literature—occasionally in the incidental sense of the anatomical distribution of lesions, and more recently, sometimes in variant form, as informal shorthand for spatial profiling of tissue [68], including a recent call to establish “spatial pathology” (in quotation marks) as a routine tool of precision oncology [69]. To our knowledge, no publication has yet defined the term. Meanwhile, the institutional landscape is fragmented: the technologies are organized under “spatial biology”, an umbrella that grew out of technology development and basic-science consortia; the European Society of Pathology’s recent expert opinion paper on the digital transformation of pathology does not yet address spatial omics [70]; and the standardization work that does exist is dispersed across immuno-oncology task forces and imaging consortia [71,72,73]. Most recently, *The American Journal of Pathology* devoted a theme issue to “spatial pathobiology”—surveying the same convergence under another banner, one centered on pathobiology but extending to diagnostic translation [74,75,76]. “Spatial pathobiology” and “spatial pathology” are complementary rather than competing labels.

Our working definition is: *spatial pathology is the branch of pathology that derives diagnostic, prognostic, and predictive information from spatially resolved measurements of tissue—morphological, molecular, and computational—treating the position of cells and molecules as primary data.* The definition is deliberately technology-agnostic and encompasses classical histopathology and immunohistochemistry.

We suggest “spathology” only as an optional shorthand, in the way “telepathology” abbreviates “pathology at a distance” [64]. Its acceptability is open to doubt. It may be misread as a typographical error for “pathology”, it is less immediately intelligible than “spatial pathology”, it may translate poorly, and, as disclosed below, it is closely associated with a company founded by the authors. If the community prefers to use only “spatial pathology”, or another term, the arguments of this article are unaffected. We invite alternative proposals and would welcome an independent consensus process on terminology.

### 5.3. Conflicts of Interest and Editorial Handling

We wish to be fully transparent. All three authors are co-founders of SPATHO AB and hold financial interests in it; SPATHO AB develops technologies and analytical methods in spatial pathology, and its name derives from the same contraction as the shorthand proposed here. Advocacy for a term associated with the authors’ company is therefore a real conflict of interest, and readers should weigh the proposal accordingly; we have tried to present the counterarguments and to make the framework independent of the shorthand. The article is a purely academic discussion: it does not describe, evaluate, or promote any product, service, or technology of SPATHO AB, presents no company data, and its arguments do not depend on the company’s existence or activities; the work received no external funding. SPATHO AB has made no commitment regarding the use of these terms, and readers should be aware that the company may seek to protect them in the future. In addition, T.H. is a Guest Editor of the Special Issue “Advances in Computational and Spatial Pathology” in which this article appears. T.H. had no role in the editorial handling of this manuscript, the selection of reviewers, or the decision on it. We also acknowledge that publication of this article may benefit the Special Issue, and readers should weigh this as well.

## 6. The Agenda a Discipline Would Need to Deliver

If the community agrees that a shared framework is worth building, the following items head the agenda.

**Consensus and scope.** A multi-stakeholder process, for example, a modified Delphi exercise including practicing diagnostic pathologists, laboratory-medicine and quality-assurance specialists, clinicians, computational scientists, industry, regulators, and patient representatives, should decide whether a distinct framework is needed, what it covers, and what it is called.

**Standardization and external quality assurance.** Head-to-head comparisons of imaging-based spatial transcriptomics platforms on serial FFPE sections reveal substantial inter-platform differences, with data quality strongly dependent on probe-panel design [59]. Consensus guidance now exists for multiplexed immunofluorescence staining, validation, image analysis, and data sharing [71,72], and minimum-information standards for multiplexed tissue images have been established [73]—but nothing yet resembles the external quality assessment schemes that made clinical immunohistochemistry reliable. Such a framework would need proficiency-testing infrastructure, reference materials, and inter-laboratory ring trials.

**Data acquisition, stewardship, and infrastructure.** Spatial experiments produce multimodal, multi-gigapixel datasets whose value depends on how they are acquired, stored, structured, and shared. Acquisition needs common metadata on tissue handling, ischemia and fixation times, panel design, and instrument settings. Stewardship needs clear provenance, consent, and access rules, including for archival clinical material, together with FAIR (findable, accessible, interoperable, reusable) practices and community-wide sharing and coordination, building on consortium data standards. Storage and processing need scalable, interoperable, and auditable pipelines: open frameworks for spatial omics data [38,63] are the beginning of what must become the equivalent of radiology’s picture archiving and communication systems (PACS) and DICOM (Digital Imaging and Communications in Medicine) infrastructure, integrated with pathology information systems rather than bolted onto them, with automated processing that is validated and version-controlled.

**Validation and clinical delivery.** Most spatial signatures remain retrospective; the exceptions—large-scale international validation [44] and prospective, biomarker-selected enrollment [49]—prove the concept but also illustrate the difficulty: even clinically validated spatial assays have struggled to achieve durable commercial and reimbursement pathways, and regulatory frameworks for laboratory-developed and AI-enabled tests remain in flux worldwide. The realistic near-term route, as recent roadmaps argue, is to use high-plex spatial discovery to define biology, then distill it into scalable, low-plex or H&E-inferred assays that fit existing laboratory economics [77], each validated analytically and clinically in its own right.

**Training.** Molecular pathology matured with societies, fellowships, and accreditation. A spatial framework would require pathologists conversant with molecular data science, and computational scientists conversant with tissue; neither is produced systematically today, and calls for such training are appearing in pathology’s own journals [74]. Fellowship tracks, core curricula, and shared vocabularies between pathology, genomics, and machine-learning communities are the necessary scaffolding.

## 7. Conclusions

Every previous re-instrumentation of pathology—the microscope, the immunostain, the sequencer, the scanner—was eventually matched by an institutional response. Spatially resolved measurement is a substantial new re-instrumentation, and a small number of spatial biomarkers have been taken to guideline mention or prospective use, but the evidence overall is heterogeneous and most spatial findings are not yet clinical tools. Whether pathology needs a distinct, named branch for this work, or whether existing bodies can absorb it, is a question that should be settled by the pathology community through an open and independent process. We offer “spatial pathology” as a description, “spathology” as an optional shorthand with the caveats and conflicts stated above, and the agenda in Section 6 as a starting point for that discussion.

## Figures and Tables

**Table 1:** Examples of spatial biomarkers and the current level of evidence.

Example	What Has Been Shown	What Has Not Been Established
Immunoscore (colon cancer) [43,44,45]	Location-aware T-cell density is prognostic in a multicenter, digitally quantified validation; guideline mention as optional prognostic adjunct	Predictive value for treatment benefit; routine use; durable commercial and reimbursement pathway
Tertiary lymphoid structures [46,47,48,49]	Retrospective association with immunotherapy benefit in several tumor types; one prospective biomarker-selected phase 2 cohort	Randomized evidence; standardized definition and scoring; generalizability across tumor types
Multiplexed IHC/IF for checkpoint blockade [50,51]	Higher pooled discrimination than single-marker or bulk biomarkers in one meta-analysis; external validation in one melanoma platform	Prospective validation; harmonized panels and analysis; freedom from publication and selection bias
Spatial transcriptomics and multiplexed imaging atlases [52,53,54,55,56,57,58]	Tissue architecture associated with outcome or progression in breast cancer	Analytical validation; cross-platform concordance [59]; clinical utility

## Data Availability

No new data were created or analyzed in this study. Data sharing is not applicable to this article.
